# Identification of hub genes correlated with tumor-associated M1-like macrophage infiltration in soft tissue sarcomas

**DOI:** 10.3389/fgene.2022.999966

**Published:** 2022-12-06

**Authors:** Minchao Lv, Feixiong He, Jinku Guo, Zhenxin Zheng, Wei Wang, Jun Xie

**Affiliations:** Department of Orthopedics, The Quzhou Affiliated Hospital of Wenzhou Medical University, Quzhou People’s Hospital, Quzhou, Zhejiang Province, China

**Keywords:** CIBERSORTx, WGCNA, soft tissue sarcoma, M1-like macrophages, tumor microenvironment, tumor immunology

## Abstract

Soft tissue sarcomas (STS) are a heterogeneous series of tumors that might result in severe disability and death. Tumor-associated M1-like macrophage infiltration plays a critical role in tumor development and progression. This study aimed at identifying the hub genes associated with M1-like macrophage infiltration in STS cells. First, the expression profiles from The Cancer Genome Atlas (TCGA) and Gene Expression Omnibus (GEO) databases were imported to calculate the level of M1-like macrophage infiltration by CIBERSORTx. Afterward, the Kaplan–Meier survival analysis was performed to evaluate the correlation between macrophage infiltration and prognosis. Then, weighted gene co-expression network analysis (WGCNA) and protein–protein interaction analysis of GEO data were applied to identify the key gene related to M1-like macrophage infiltration, followed by the functional analysis using TCGA cohort to validate downstream signaling associated with the gene. Finally, pan-cancer analysis was conducted to investigate the gene function in other types of tumors. We found LCK expression positively related to the M1-like macrophage infiltration level, and it positively regulated the expression level of genes regulated to macrophage polarization, and chemotaxis, including interferon-γ (INF-γ), interleukin-12 (IL12), tumor necrosis factor (TNF), PI3K, NF-κB, and CXCL9, 10, and 11. In summary, an ‘LCK-INF-γ/IL-12-TNF/PI3K-NF-κB’ axis might exist in STS cells that regulate M1-like macrophage infiltration.

## Introduction

Soft tissue sarcomas (STS) are a heterogeneous series of tumors derived from mesenchymal tissue, which account for approximately 1% of solid tumors (Mohindra and Agulnik, 2015). This series include over 50 different histologic subtypes that are heterogeneous in terms of anatomical location, molecular characteristics, and prognosis (Tseng et al., 2014; Yen and Chen, 2018). The most common anatomic sites of STS in the human body are the extremities (60–70%) and the abdomen and retroperitoneum (20%) (Tseng et al., 2014). It is reported that STS with highly aggressive behavior was responsible for more than 5,000 deaths and much more patients with permanent disability in 2019 despite its lower incidence than common cancers (Siegel et al., 2019). Owing to its early metastatic characteristics, STS are usually inoperable when diagnosed, which makes chemotherapy the first-line therapy in clinics (Yen and Chen, 2018; Ruiu et al., 2019). However, the high recurrence rate of STS and the severe systematic toxicity of traditional chemotherapeutics let the overall prognosis far lower than people expected (Sousa et al., 2021). Therefore, the treatment of STS is still a tough challenge to most clinicians around the world.

Recently, the success of tumor immunotherapy, especially checkpoint blockade therapy both in laboratory research and clinical trials, has revealed the dominant role of tumor-infiltrating immune cells in tumor growth and progression (Miller et al., 2019). The macrophage, a kind of vital innate immune cell located in tissues, which possibly plays a role in tumor development and progression, has been reported (Aras and Zaidi, 2017). Tumor-associated macrophages (TAMs) are major components in the tumor microenvironment (TME) and play a critical role in tumor-related inflammation (Noy and Pollard, 2014; Mantovani et al., 2017; Yang and Zhang, 2017). In general, TAMs are differentiated by the TME into M1-like or M2-like macrophages, which typically display an anti-tumorigenic function or a pro-tumorigenic function, respectively (Malfitano et al., 2020). M1-like macrophages are capable of presenting antigens, secreting interleukin and nitric oxide, activating type I T-cell response, and subsequently inhibiting cell proliferation, as well as causing tissue damage (Verreck et al., 2004; Bouhlel et al., 2007; Allavena et al., 2008). Moreover, TAMs are also involved in angiogenesis and lymphangiogenesis, which form the structure basis of tumor development and metastasis (Gordon et al., 2010; He et al., 2016). These tumor-associated effects make M1-like TAMs the potential therapeutic target in the treatment of STS. It has been reported that extrinsic polarization, which is mediated by the cytokines, HIF1/2, and endothelin-2 secreted from other immune cells, was the primary method of macrophage polarization (Ivashkiv, 2020; Boutilier and Elsawa, 2021). Phenomenally, the polarization of macrophages highly correlated with tumor cells within the TME (Mosser and Edwards, 2008). However, few studies focused on the molecular mechanisms of the interaction between soft tissue tumors and M1-like macrophages.

Herein, bioinformatic tools were applied to evaluate the association between M1-like macrophage infiltration and prognosis of STS and identify the hub genes potentially associated with M1-like macrophage infiltration in STS. Moreover, the interested hub gene was validated in STS cases from The Cancer Genome Atlas (TCGA) cohort. Finally, the association between the interested hub gene and M1-like macrophage infiltration in other cancer cases from TCGA and GTEx cohorts was also evaluated.

## Materials and methods

### Patients and samples

Gene expression data on 9, 549 samples of 39 types of cancers (including 265 STS patients from TCGA-SARC cohort) from TCGA database were downloaded *via* Xena (https://xenabrowser.net/datapages/). Raw gene expression data from the GSE21122 dataset, including 149 STS samples and 9 control normal fat samples, were also downloaded from the Gene Expression Omnibus (GEO) database. Subsequently, the raw gene expression profiles of GSE21122 were preprocessed by the “RMA” algorithm (Gautier et al., 2004). During the probe mapping process, mean values were maintained if multiple probes shared the same gene symbol. In addition, clinical data of TCGA and GEO samples were also downloaded.

### Evaluation of M1-like macrophage infiltration

In this work, CIBERSORTx (Stanford University) (Newman et al., 2019), a digital cytometry tool that enables researchers to estimate the fraction of 22 kinds of immune cell infiltration *via* gene expression profiles from bulk tissue, was applied to evaluate the tumor-associated M1-like macrophage infiltration in STS tissue using the data from TCGA-SARC cohort and the GSE21122 cohort. Samples with a p-value less than 0.01 in CIBERSORTx calculation results were selected for further analysis.

### Weighted gene co-expression network analysis and visualization

The WGCNA R package was applied to construct the gene co-expression network, according to the gene expression profiles of the GSE21122 dataset (Barretina et al., 2010). A total of 149 tumor tissue samples in the GSE21122 dataset were included for further analysis. First, the unqualified genes and samples were excluded *via* the goodSamplesGenes method in the WGCNA R package. Then, a weighted adjacency matrix was constructed *via* calculating the Pearson correlation between all pairs of genes. After that, the power of *β* = 5 (scale-free *R* ^2^ = 0.87) was used as a soft thresholding parameter to construct the scale-free network. The adjacency matrix was then transformed to a topological overlap matrix (TOM) to further identify functional modules in the co-expression network. To classify genes with similar expression profiles into gene modules, average linkage hierarchical clustering was conducted, according to the TOM-based dissimilarity measure with a minimum size (gene group) of 30 and a sensitivity of 3, for the gene dendrogram.

The module eigengenes (MEs) were described by principal component analysis, corresponding to a single characteristic expression profile of all genes in each module. In order to identify which module was most clinically significant, the correlation between these eigengenes and the clinical characteristic (macrophage M1 score) was calculated and described as a gene significance (GS), equal to the logarithm of the p-value for the individual gene. The candidate hub genes were considered as the genes whose GS strongly correlated with the module membership (MM), defined as the correlation between individual gene expression profiles and the module’s eigengenes.

Subsequently, all identified candidate hub genes were imported into the STRING database to perform protein–protein interaction (PPI) analysis (Szklarczyk et al., 2019). The analysis results were visualized *via* the PPI network image.

### Differentially expressed gene analysis and gene set enrichment analysis in TCGA cohort

The expression data on TCGA-SARC cohort were downloaded, divided equally into two groups according to the expression level of the candidate hub gene, and analyzed by the R package limma (version 3.40.6), a gene differential expression screening method based on the generalized linear model, to identify differentially expressed genes. All genes were subsequently arranged in order by log_2_(FoldChange). The ordered gene list was then imported for GSEA analysis by GSEA software (version 3.0, Broad Institute).

### Cell culture

The human rhabdomyosarcoma cell line A-673 (ATCC CRL-1598) was purchased from BeNa Culture Collection (Beijing, China). A-673 cells were cultured in Dulbecco’s modified Eagle’s medium with high glucose (DMEM-H) (Gibco, USA) supplemented with 10% (v/v) fetal bovine serum (FBS) (Gibco, USA) and 1% (v/v) penicillin/streptomycin (Beyotime, Shanghai, China). The cells were maintained in an incubator at 37°C with 5% CO_2_. The medium was replaced every 2 days during the culture.

### LCK knock-down and quantitative polymerase chain reaction assay

A-673 cells were seeded in a 6-well plate at a density of 1 × 10^5^ cells per well and cultured at 37°C with 5% CO_2_ for 24 h. Then, the cells were transfected with Lck-specific FAM-siRNA (Ruibo Biotech, China) for 48 h using Lipofectamine 2000 (Invitrogen, USA), following the manufacturer’s instruction. The transfection efficiency was evaluated with the fluorescence intensity measured using an inverted fluorescence microscope (ZEISS, Germany) (at 6 h after the addition of siRNA) and the LCK expression level measured by qPCR.

For qPCR assay, total RNA was isolated and purified using the TRIzol reagent (Invitrogen, USA), following the manufacturer’s instruction. The total RNA concentration and purity of each sample were quantified using a NanoDrop ND-1000 spectrophotometer (NanoDrop, USA). Then, cDNA was synthesized from 1 μg of the total RNA sample by reverse transcription with the PrimeScript™ RT Master Mix (TaKaRa, Japan). qRT-PCR was conducted on a Roche LightCycler 480 Instrument II (Roche, USA) with TB Green ^®^ Premix Ex Taq™ (TaKaRa, Japan). The primer sequences used in the qRT-PCR are shown in Supplementary Table S1. Data were analyzed using the comparative Ct method (2^−ΔΔCt^).

### Statistics

All statistical analyses were performed by R and GraphPad software applications. For continuous variables, the unpaired t-test or one-way analysis of variance (ANOVA) was applied to evaluate the statistical significance between normal and tumor groups. The Kaplan–Meier method and log-rank test were performed to evaluate and illustrate the differences of survival between high and low M1-like macrophage infiltration groups. The cutpoint to determine high/low M1-like macrophage infiltration was calculated *via* the R package MaxStat (maximally selected rank statistics with several p-value approximations). Data visualization was performed based on the R packages ggpubr and ggplot2.

## Results

### Study design

**FIGURE 1:**
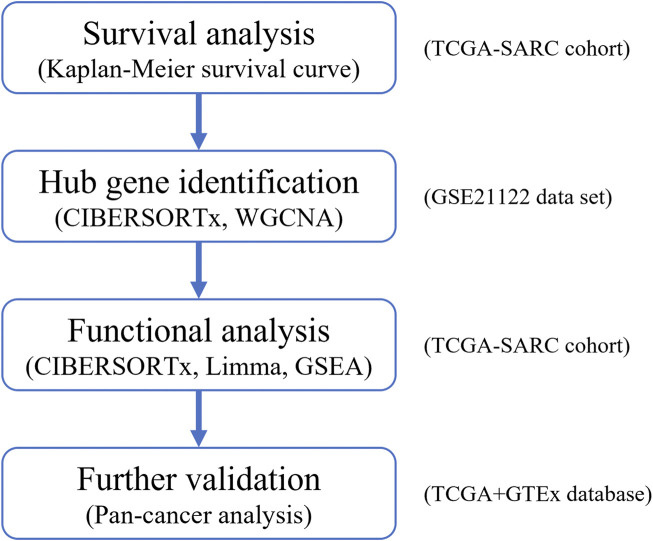
Study design.

The design of this work is illustrated in Figure 1. The gene expression profiles from the GSE21122 dataset (n = 158) and TCGA-SARC cohort (n = 265) were imported into CIBERSORTx to calculate for macrophage M1 scores. Following identifying the hub genes associated with M1-like macrophage infiltration with GEO data, the correlation between the candidate hub genes and M1-like macrophage infiltration in various kinds of tumors was validated using TCGA and GTEx databases. Finally, the functional analysis of downstream signaling connecting with the candidate hub gene expression and tumor-associated M1-like macrophage infiltration was performed.

### High tumor-associated M1-like macrophage infiltration induced better prognosis of STS patients

**FIGURE 2:**
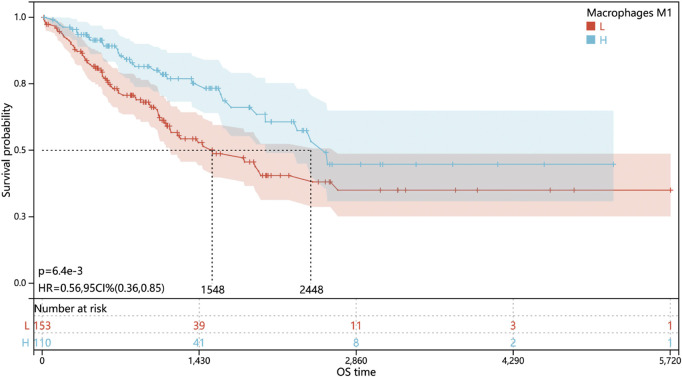
Level of M1-like macrophage infiltration associated with better prognosis in soft tissue sarcomas. Kaplan–Meier overall survival curves of STS patients in TCGA cohort stratified by a low or high M1-like macrophage infiltration level.

Since the lack of prognosis data, especially survival data on the GSE21122 dataset, only the data from TCGA-SARC cohort were subjected for analysis of the correlation between the M1-like macrophage infiltration level and the prognosis of STS patients. The results (Figure 2) showed that high M1-like macrophage infiltration indicated better prognosis in STS patient (*p* < 0.05), which highlighted the clinical relevance of tumor-associated M1-like macrophage infiltration and the urgency to investigate the molecular mechanism of the M1-like macrophage–tumor interaction in soft tissue sarcomas.

### Identification of hub genes associated with M1-like macrophage infiltration *via* WGCNA analysis of the GEO dataset

**FIGURE 3:**
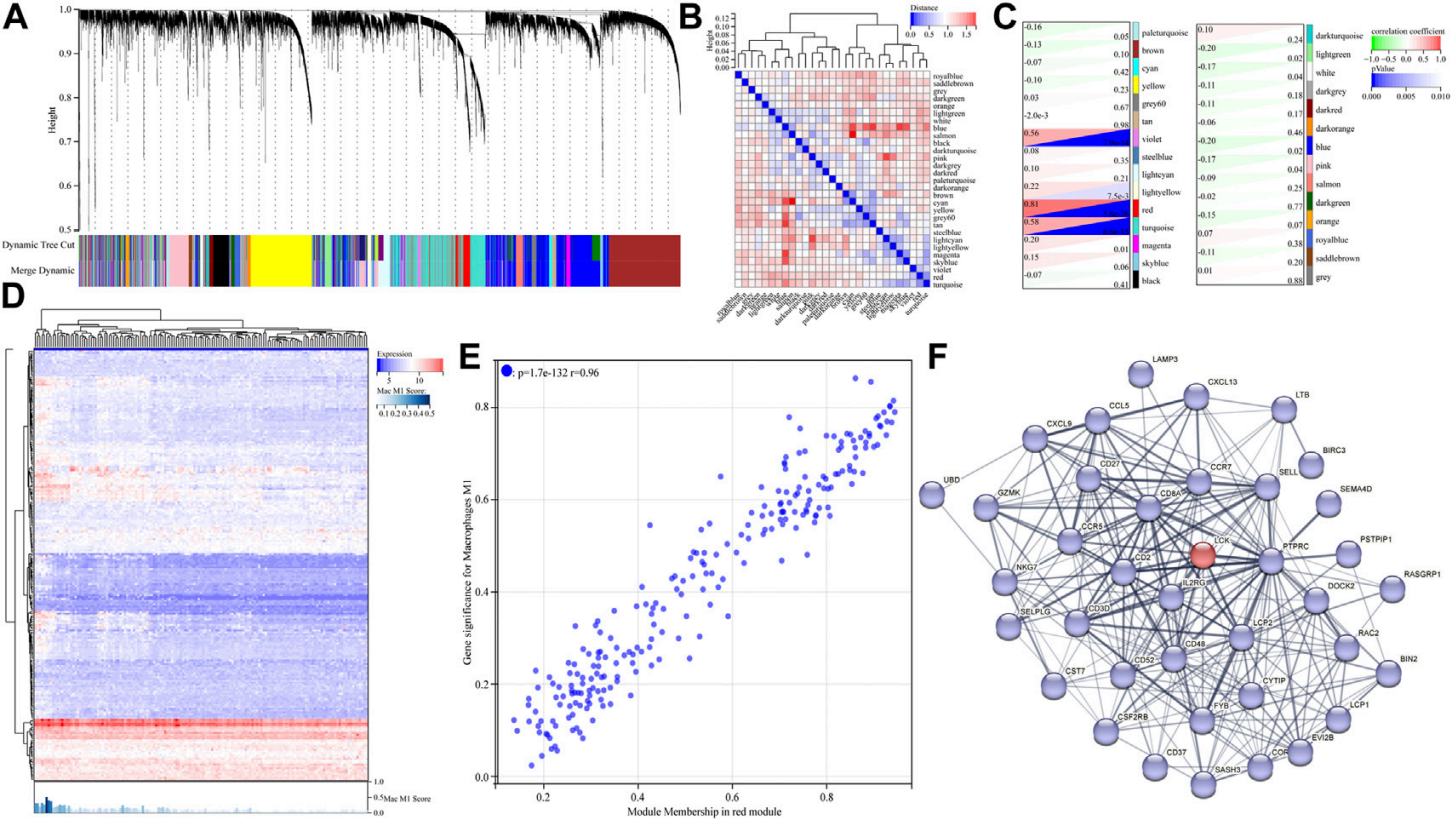
Identification of hub genes correlated with M1-like macrophage infiltration in the GSE21122 dataset. **(A)** Dendrogram of all differentially expressed genes clustered based on a dissimilarity measure. Dynamic tree cutting was applied to identify modules by dividing the dendrogram at significant branch points. **(B)** Heatmap of the adjacencies of modules along with the module trait relationship. **(C)** Correlation between the expression of the gene module and clinical characteristic (M1-like macrophage infiltration). **(D)** Heatmap of the expression level of genes in the red module obtained from WGCNA analysis. **(E)** Gene significance–module membership (GS-MM) scatter diagram of genes in THE red module. **(F)** Protein–protein interaction analysis of all candidate hub genes in the red module, *via* the STRING database.

First, we filtered out the tumor samples in the GSE21122 data set with p-values (derived from CIBERSORTx calculation) greater than 0.01 to ensure the robustness of WGCNA. A total of 149 tumor tissue samples were finally imported to WGCNA analysis. The power of *β* = 5 (scale-free *R* ^2^ = 0.87) was selected as a soft thresholding parameter to construct the scale-free network (Supplementary Figure S1). A total of 29 gene modules represented by 29 different colors were successfully identified (Figures 3A,B). After that, the correlation between the expression of each gene module and the clinical characteristic (macrophage M1 score) was calculated and visualized, as shown in Figures 3C,D. As shown, the red module most significantly correlated with the macrophage M1 score (with the lowest p-value and the highest correlation coefficient). The GS-MM scatter diagram (Figure 3E) also illustrated that the expression level of genes in the red module highly correlated with the level of tumor-associated M1-like macrophage infiltration (r = 0.96; *p* = 1.7 × 10^−132^). As expected, a series of macrophage polarization markers on human M1-like macrophages, including CXCL9, 10, and 11 (Boutilier and Elsawa, 2021), could be identified in the red module, which reflected the robustness of WGCNA. The genes in the red module with a GS value greater than 0.1 and an MM value greater than 0.8 were filtered and considered candidate hub genes. Finally, a total of 41 hub genes were screened, and the whole list is shown in Supplementary Information.

To further explore the interactions between the hub genes mentioned previously, the protein–protein interaction analysis of all candidate hub genes in the red module was performed and is given in Figure 3F, *via* the STRING database (the disconnected nodes were hidden in the network). As shown, LCK, a proto-oncogene and a member of the Src family of protein tyrosine kinases, interacted with much of hub genes in the PPI network. Therefore, we assumed that LCK potentially induced M1-like macrophage infiltration in tumor tissue *via* specific downstream signaling pathways. Moreover, the 10 best interactors influenced by LCK are provided as follows with their interacting scores: PTPRC (0.999), CD8A (0.999), LCP2 (0.998), CD2 (0.991), CD3D (0.988), CD48 (0.954), IL2RG (0.949), FYB (0.617), RASGPR1 (0.576), and CD27 (0.566). These interactors might provide some support in finding the new markers of M1-like macrophages, which needs further investigation.

### Functional analysis of downstream signaling associated with LCK expression

**FIGURE 4:**
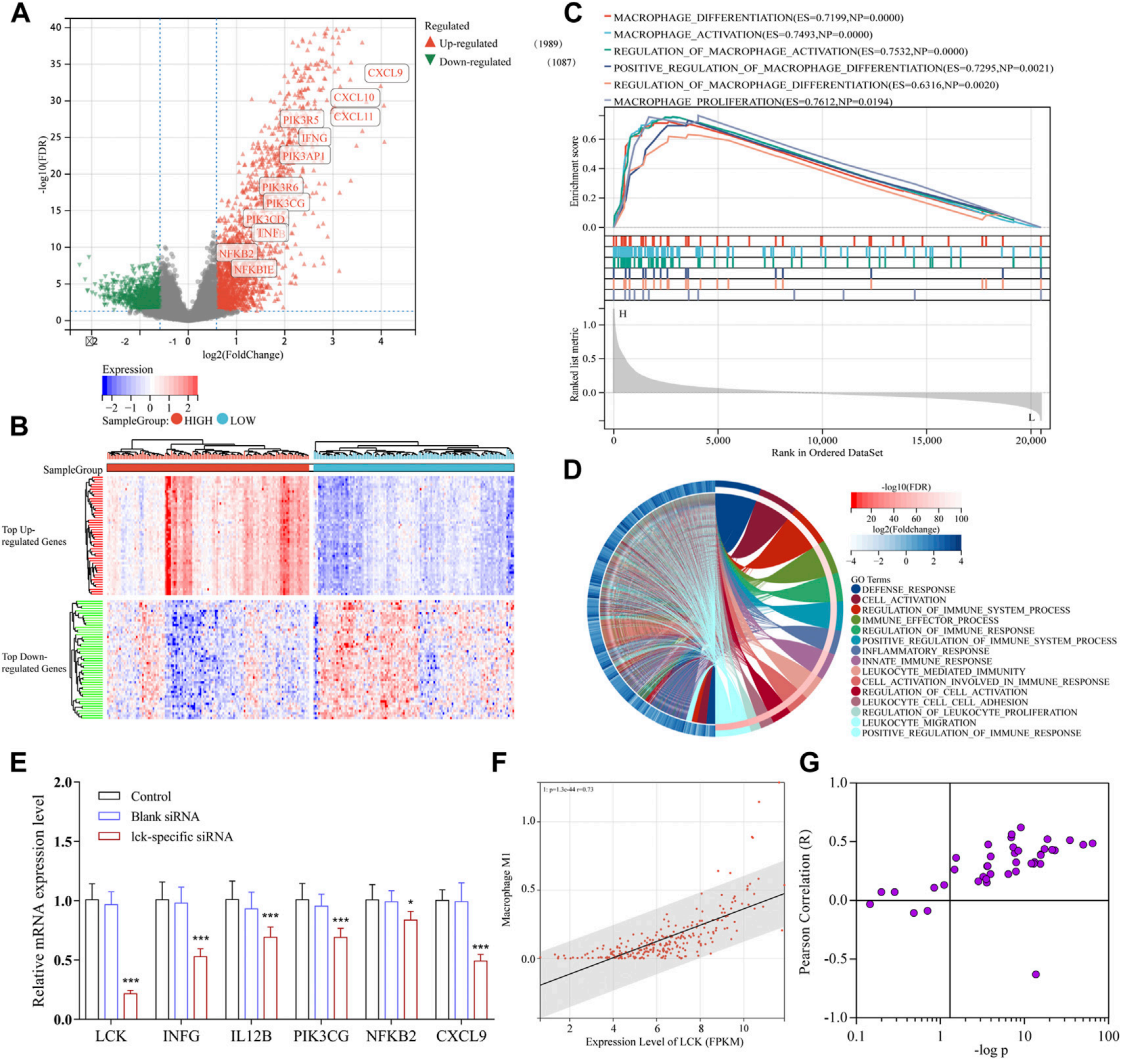
Functional analysis of downstream signaling associated with LCK expression in TCGA-SARC cohort. **(A)** Volcano plot of differentially expressed genes in the gene expression profiles between high- and low-LCK groups. There were 1989 upregulated genes and 1,087 downregulated genes among 20,475 genes in expression profiles. **(B)** Heatmap of top 25 upregulated and downregulated genes between high- and low-LCK groups. **(C)** GSEA analysis of the GO biological process. **(D)** GO biological process enrichment analysis of differentially expressed genes between LCK-low and -high groups. **(E)** PCR results of key genes in the LCK-involved regulatory axis of TAM infiltration in A-673 cells transfected with and without LCK-specific siRNAs. The correlation between the expression level of LCK and level of M1-like macrophage infiltration in STS **(F)** and other 44 types of solid tumors **(G)**.

TCGA-SARC cohort was used to further validate and investigate the LCK-associated biological progress. Based on the expression level of LCK, the samples were divided into two groups, with 132 and 133 samples in LCK-high and -low groups, respectively. Differentially expressed genes were calculated and is given in Figures 4A,B. The heat map illustrated that LCK expression significantly influenced a series of genes. As shown in the volcano plot, the expression levels of genes encoding interferon-γ (*INFG*), interleukin-12 (*IL12B*), PI3K (including *PIK3R5*, *PIK3AP1*, *PIK3CG*, *PIK3CD*, and *PIK3R6*), and NF-κB (including *NFKB2* and *NFKBIE*), as well as a series of polarization markers on M1-like macrophages (including *CXCL9*, *10*, and *11*), were significantly higher in the LCK-high group than that in the LCK-low group. Gene set enrichment analysis (GSEA) of the GO biological process (Figure 4C) and GO biological process enrichment analysis (Figure 4D) suggested the expression of LCK significantly influenced M1-like macrophage differentiation, activation, and polarization. These findings indicated that an LCK-involved tumor-associated M1-like macrophage differentiation/polarization axis might exist.

To further confirm the existence of the LCK-involved regulatory axis in tumor-associated M1-like macrophage infiltration, LCK gene expression was knocked down in A-673 cells (the human rhabdomyosarcoma cell line) by LCK-specific FAM-siRNA. As shown in Supplementary Figure S2, the LCK expression was desirably knocked down in siRNA-transfected cells. Then, qPCR assay was applied to measure the gene expression level of interferon-γ (*IFNG*), interleukin-12 (*IL12B*), PI3K (*PIK3CG*), NF-κB (*NFKB2*), and CXCL9 in LCK-knocked down cells. As shown in Figure 4E, the gene expression levels of *INFG*, *IL12B*, *PIK3CG*, *NFKB2*, and CXCL9 were significantly decreased in LCK-knocked down cells, which was consistent with the bioinformatic analysis.

### Validation of LCK-associated M1-like macrophage infiltration in TCGA and GTEx cohorts

To further validate the correlation between LCK expression and tumor-associated M1 macrophage infiltration in other types of tumors, the standardized gene expression profiles of 44 types of solid tumors with 10,173 tumor samples from TCGA and GTEx cohorts were subjected to CIBERSORT calculation.

As shown in Figure 4F, in STS patients, the expression level of LCK significantly correlated with tumor-associated M1-like macrophage infiltration (*p* = 1.33 × 10^−44^; r = 0.73). Figure 4G illustrated the correlation between the LCK expression level and M1-like macrophage infiltration in other kinds of solid tumors, which indicated that in a large proportion of tumor types, LCK was significantly associated with M1-like macrophage infiltration.

**FIGURE 5:**
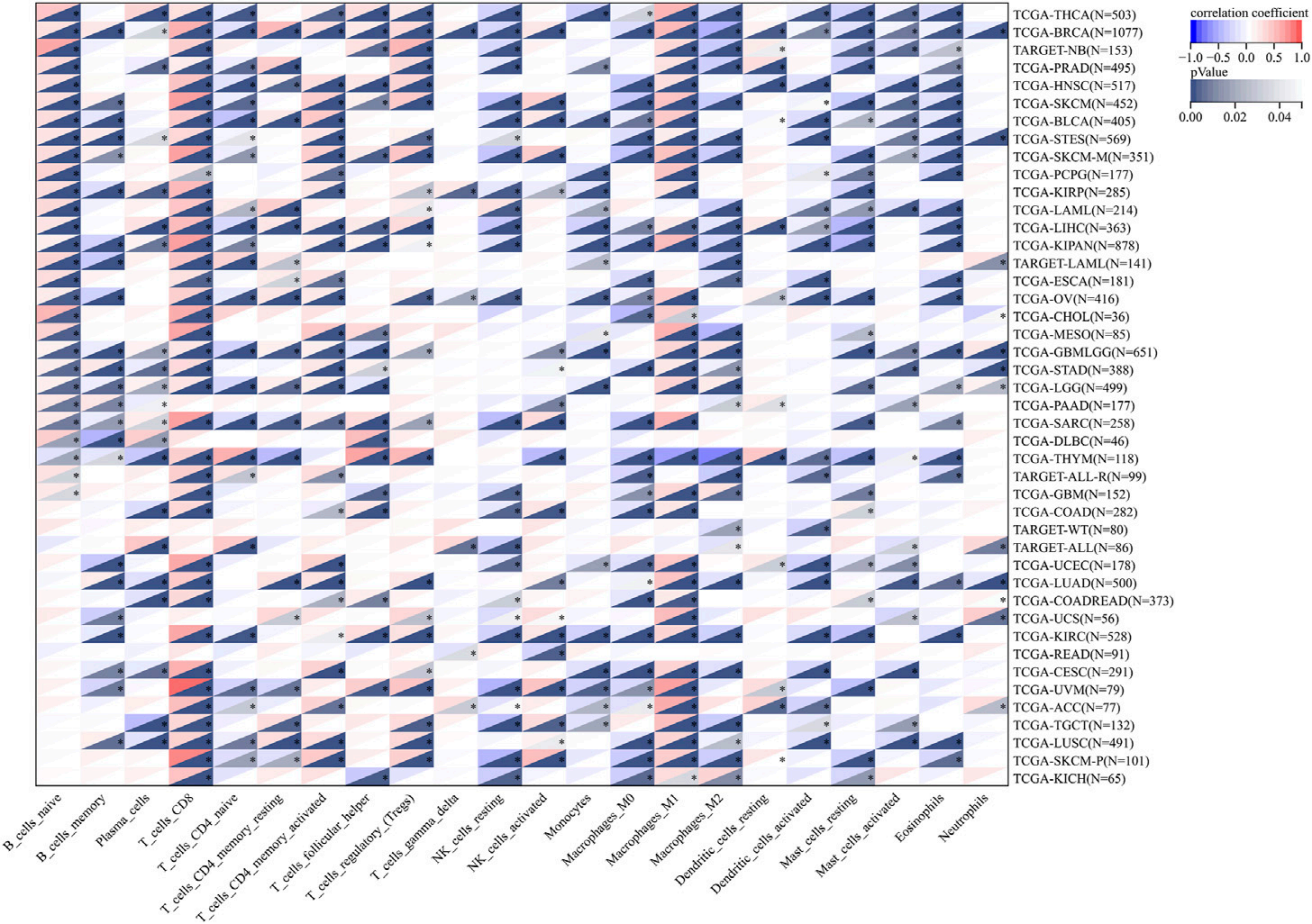
Correlation between the expression level of LCK and 22 types of immune cell infiltration in 44 types of solid tumors. The original gene expression profile data were derived from TCGA and GTEx databases.

To further verify the role of LCK in tumor-associated immune cell infiltration, the correlation between the LCK expression level and 22 kinds of immune cell infiltration scores in 44 types of solid tumors were calculated by CIBERSORT and is visualized in Figure 5. As shown, LCK most highly correlated with the infiltration of M1-like macrophages and CD8^+^ T cells, which indicated LCK played an important role in tumor-associated immune cell infiltration in most solid tumors.

## Discussion

Owing to the remarkable success of immune checkpoint inhibitor (ICI) therapy in clinical trials, anti-tumor immunotherapy has been put under the spotlight in recent decades (Miller et al., 2019). However, a large proportion of patients (>70%) showed low sensitivity to ICIs in clinics, which suggests the necessity to figure out alternative therapeutic targets.

Tumor-associated M1-like macrophages are validated as a kind of essential tumor-suppressing cells (Fan et al., 2006). In the TME, M1-like macrophages are capable of recruiting and activating CD8^+^ T cells and NK cells *via* antigen presentation to the T-cell receptor and the secretion of tumor-derived chemokines including CXCL9, CXCL10, and CXCL11 (Hadrup et al., 2013; Susek et al., 2018). Subsequently, the cytokines and chemokines secreted by CD8^+^ and NK cells assisted in further signaling of anti-tumorigenic pathways and recruitment of immune cells (Fan et al., 2006; Mosser and Edwards, 2008). Moreover, the CXCL9, 10, or 11–CXCR3 ligand/receptor pair has also been reported to be downregulated in the metastatic and therapy-resistant tumor, which suggested the upregulation of CXCL9, 10, and 11 might be potential therapy targets for recurrent tumors (Szekely et al., 2018). In summary, tumor-associated M1-like macrophages might be a promising therapeutic target for STS and value further exploration in STS therapy.

As tumor-associated macrophages play an essential role in tumor development, progression, metastasis, and angiogenesis, it could also be used to predict the clinical outcome. Lee et al. revealed that the high density of both M1-like and M2-like macrophage infiltration significantly associated with poor prognosis of non-gynecologic leiomyosarcomas (Lee et al., 2008). Kostine et al. figured out only M2-like macrophage infiltration highly correlated with poor prognosis of leiomyosarcomas (Kostine et al., 2018). However, research studies focused on the correlation between the prognosis of STS and M1-like macrophage infiltration still remain insufficient to establish consensus. In this study, Kaplan–Meier survive analysis was applied in TCGA-SARC cohort. The results revealed that M1-like macrophage infiltration positively and significantly correlated with better prognosis in STS patients, which also suggested the necessity to explore the mechanisms behind M1-like macrophage infiltration in STS.

Extrinsic polarization is a primary macrophage polarization method mediated by cytokines secreted by other cells in the TME (Boutilier and Elsawa, 2021). It was validated that extrinsic interferon-γ (INF-γ) and interleukin-12 (IL-12) drove the polarization of macrophages toward the M1 phenotype (Bouhlel et al., 2007; Cohen and Mosser, 2013), *via* several of various signaling pathways, including the LPS-TLR4-PI3K-AKT-mTORC1 axis (Arranz et al., 2012; Covarrubias et al., 2015), AKT2-NF-κB activation axis (Fukao and Koyasu, 2003), and TNF-TNFR-NF-κB activation axis (Kratochvill et al., 2015). In our work, it has been proved that the upregulated expression of LCK induced a higher expression level of genes encoding INF-γ, IL-12, TNF, PI3K, and NF-κB. Moreover, LCK influenced the processes of M1-like macrophage differentiation, activation, and polarization, according to the GSEA result. All these results reached a conclusion that there might be an ‘LCK-INF-γ/IL-12-TNF/PI3K-NF-κB’ axis existing in STS cells that regulated M1-like macrophage polarization. In addition, higher expression of LCK has been confirmed in this work that it also upregulated the expression levels of CXCL9, CXCL10, and CXCL 11, which were reported to initiate a chemokine network to contribute to the generation of a “hot” (or T-cell–inflamed) tumor microenvironment (Reschke and Gajewski, 2022). Researchers also figured out the combination therapy of CXCL9/10 agonists and immunotherapy drugs improved the overall response rate in metastatic melanoma (Ribas et al., 2021) and epithelial ovarian cancer (Orr et al., 2022). However, the aforementioned agonists of CXCL9/10 worked efficiently only when critical myeloid cells (M1-like macrophages and dendritic cells) presented within the tumor microenvironment (Hoch et al., 2022; Reschke and Gajewski, 2022). In summary, this study revealed that the upregulated expression of LCK might not only induce the polarization of tumor-associated macrophages toward the M1 type *via* the ‘LCK-INF-γ/IL-12-TNF/PI3K-NF-κB’ axis but also improve the secretion of CXCL9, 10, and 11 in STS microenvironments, which work together to improve the overall response rate and prognosis of STS patients.

LCK is a member of the Src family of protein tyrosine kinases (PTKs), located in the plasma membrane and pericentrosomal vesicles and highly expressed in immune organs including the lymph node and appendix. The encoded protein is known as a key signaling molecule in maturation of developing T cells, which are a kind of key cells in tumor immunology (Wu et al., 2021). Interestingly, except for being associated with CD8^+^ T-cell infiltration, we found that the expression level of LCK also positively correlated with M1-like macrophage infiltration in most kinds of solid tumors, indicating a universal association between LCK and M1-like macrophage infiltration in the TME, which also suggested additional functions of LCK in tumor progression except for regulating T-cell maturation.

However, there were some inherent limitations in the validity of bioinformatic analysis as the accuracy of conclusion was highly dependent on the quality of gene expression profiles and phenotype data subjected in the analysis. In this work, the conclusion was validated twice by the GEO data set (n = 149) and TCGA-SARC cohort (n = 265), to ensure the validity as far as possible. Moreover, as there were more than 50 different histologic subtypes in STS, the bioinformatic analysis, without distinguishing the histologic subtypes, may limit the validity of the analysis.

Altogether, we suggested that an ‘LCK-INF-γ/IL-12-TNF/PI3K-NF-κB’ axis might exist in STS cells that regulate M1-like macrophage infiltration. Moreover, the activation of LCK also improved the secretion of CXCL9, 10, and 11, which work together with M1-like macrophages to improve the overall response rate and prognosis of STS patients. The finding in this work provided insights into the molecular mechanisms of crosslinks between soft tissue sarcomas and M1-like macrophage infiltration, which potentially needs to be further investigated by clinical cohorts and might facilitate further investigation of tumor-associated M1-like macrophage infiltration in the future.

## Conclusion

Herein, a list of hub genes highly correlated with tumor-associated M1-like macrophage infiltration were filtered out based on the comprehensive analysis of gene expression profiles using CIBERSORTx and WGCNA algorithms. Among these, LCK was significantly associated with M1-like macrophage infiltration and cytokines, including INF-γ, IL-12, and TNF, which drove the polarization of macrophages toward the M1 phenotype. These results indicated the potential existence of the ‘LCK- INF-γ/IL-12-TNF/PI3K-NF-κB’ axis that regulated M1-like macrophage infiltration in the STS microenvironment. Meanwhile, LCK also improved the secretion of CXCL9, 10, and 11, which improved the overall response rate and prognosis of STS patients with the help of M1-like macrophages. The findings of this work might facilitate further investigation in crosstalk between tumor-associated M1-like macrophages and STS cells in the future.

## Data Availability

The original contributions presented in the study are included in the article/Supplementary Material; further inquiries can be directed to the corresponding author.
