# Supplementary material for: Identification of hub genes correlated with tumor-associated M1-like macrophage infiltration in soft tissue sarcomas
Source: Front Genet. 2022 Dec 6;13:999966. doi: 10.3389/fgene.2022.999966 (PMC9763622; doi:10.3389/fgene.2022.999966)
Supplement: Supplementary file 1 [file DataSheet1.docx]

**Identification of Hub Genes Correlated with Tumor-associated M1-like Macrophage Infiltration in Soft Tissue Sarcomas**

Minchao Lv^1^, Feixiong He^1^, Jinku Guo^1^, Zhenxin Zheng^1^, Wei Wang^1^#, Jun Xie^1^#.

**Supplementary Information**

Number of Figures: 2

Number of Tables: 1

**List of hub genes in WGCNA analysis**

ADA2; LCP1; CXCL9; CD48; TRBC1; IL2RG; GZMK; CCR5; CSF2RB; CD3D; RAC2; PTPRC; CORO1A; SELPLG; CD2; CD8A; SASH3; LTB; CCL5; FYB1; SEMA4D; UBD; CD27; SELL; CST7; CD52; DOCK2; CD37; BIN2; LCP2; EVI2B; PSTPIP1; NKG7; LCK; CXCL13; LAMP3; CYTIP; RASGRP1; GPR171; CCR7; BIRC3.

**Table S1**. Primer sequences for quantitative polymerase chain reaction (qPCR) assay.

| Gene | Forward Sequence | Reverse Sequence |
| --- | --- | --- |
| β-actin | 5’‐CTGGAGCATGCCCGTATTTA‐3’ | 5’‐TTTGGTCTTGCCACTTTTCC‐3’ |
| INFG | 5’-TCGGTAACTGACTTGAATGTCCA-3’ | 5’-TCGCTTCCCTGTTTTAGCTGC-3’ |
| IL12B | 5’-GCGGAGCTGCTACACTCTC-3’ | 5’-CCATGACCTCAATGGGCAGAC-3’ |
| PIK3CG | 5’-GGCGAAACGCCCATCAAAAA-3’ | 5’-GACTCCCGTGCAGTCATCC-3’ |
| NFKB2 | 5’-ATGGAGAGTTGCTACAACCCA-3’ | 5’-CTGTTCCACGATCACCAGGTA-3’ |
| CXCL9 | 5’-CCAGTAGTGAGAAAGGGTCGC-3’ | 5’-AGGGCTTGGGGCAAATTGTT-3’ |
| LCK | 5’-TGCCATTATCCCATAGTCCCA-3’ | 5’-GAGCCTTCGTAGGTAACCAGT-3’ |


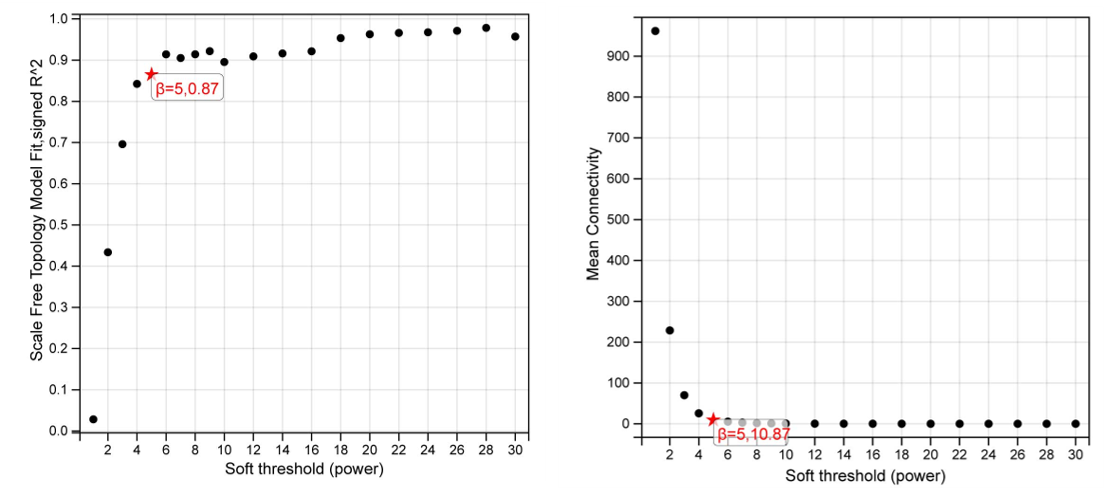


**Fig. S1**. Analysis of (A) the scale-free fix index and (B) the mean connectivity for various soft-thresholding powers in WGCNA analysis.


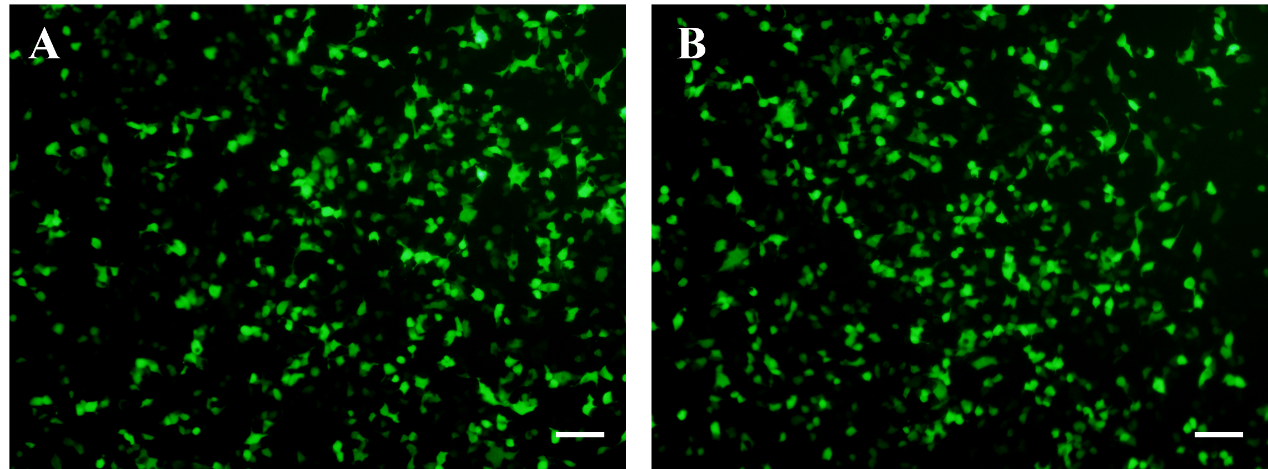


**Fig. S2.** Fluorescence images of A-673 cells transfected with lck-specific eGFP-siRNA (A) and blank eGFP-siRNA (B) via lipofectamine 2000. Scale bar equals to 200 μm.
